# Oldboy Brother’s Nearest Relative

**Published:** 1876-11

**Authors:** Charles H. Ross


### *OLDBOY BROTHERS' NEAREST RELATIVE:*

### A TOOTLETUM TALE WITH A SENTIMENTAL ENDING.

BY CHARLES H. ROSS.

I AM Oldboy Brothers.

Properly speaking, though, I am a Child. That is to say I am a Child by name. In years I'm properly an old boy. Not wishing, however, to be more confusing than is absolutely necessary at this early period of my story, I may as well mention that the two old men who were the original Oldboy Brothers, Barbadoes, died intestate, leaving a fortune behind them, and that I, their head clerk, carried on the business till I had made a fortune also, and that I carried it on in the name of Oldboy Brothers. It doesn't matter to me very much whether you go on calling me Oldboy Brothers or not. When you come to be called out of your name for a matter of forty odd years, to be called in your name again is rather a trouble to you than otherwise. As, however, it may be necessary that I should be known as something or other decisively, let's say Oldboy

Brothers once for all, and stick to it.

Well, then, Oldboy Brothers' nearest relative is a Man, and (here we are getting confusing again) that Man is a female. You see she is only called Man. Ann Man is her right name. She is a kind of aunt, and I came over from Barbadoes to leave her my fortune.

This again, on the face of it, is seemingly getting more confusing than ever. A man doesn't generally make a will in favor of his maiden aunt, who may be not unnaturally supposed to be considerably his senior; but, you see, there was no one in Barbadoes that I particularly took to, and not having any children of my own I wanted to leave my money to the Childs generally, if any were alive. Inquiries proved that the race of Childs I used to belong to was extinct, with the exception of my aunt Man, so I made my mind up to come back to England and find her out, and make her take my money if I died first.

I may as well as say, before I go further, that the Childs were never a particularly affectionate family. If you search the archives of the Chancery Courts, you will find the case of Child *v.* Child occupying space, and there have been pretty nearly as many Childs divorced as there have been Smiths. When I was fifteen it was war to the knife between me and my relations, and that was why I went to Barbadoes. When I was forty-five and had made my money, I came back again, thinking to find a Child or two left. The Kilkenny cats were, comparatively speaking, quite sociable compared with the Childs. They must have fought hard, though, and died game, for some of them were plaguey tough.

Nearly one of the plagueyest toughest among them was my maternal grandfather, who buried four wives, and died a lonely widower after all, about eighty odd years of age. It was his surviving daughter, Ann Man, who was my last remaining relative on earth, and she lived a long way off in the country.

The nearest railway station is eighteen miles from the house my aunt lived at, which was a house standing all by itself in the middle of fields, and to be got at only by circuitous roads indescribably rutty. The flyman I sent for from the station (he came four miles across country in answer to my summons to take me up) pointed out a near cut when we had jolted on a couple of hours or so, and said: "Yon's t' nighest road, cross they turnips and round t' croft agin t' 'ay-stack, if ye choosen to git out an' walk."

I thought I chose to do so, as I ached a good deal, and I paid his fare and scrambled through a gap he pointed out in the hedge.

From the fly-window I could see plainly enough the gable ends of the old red brick house Miss Ann Man lived in, but the turnip-field took a sudden dip about the middle, and here I lost my bearings, and came out at the opposite end to that I had been directed to, opposite a five-bar gate, with a man on the topmost bar with his mouth full of bread and cheese.

I said, "I've lost my way."

He said, "I sh'd think thee had, mun. Tha'll a t' mussus abart thee ears if thee darnt watch it."

I said, "Do you belong to Miss Man's farm?"

He said, "A reckon so."

I said, "I want to go there, then. Will you show me the way? I'm Miss Man's nephew."

He burst into such a guffaw on receipt of this intelligence, that a wedge of bread and cheese went the wrong way and nearly choked him.

"Tha' meanst to say, mon, tha'rt Miss Ann's nevvy? Ga 'long wi' thee! Thar't chaffin' loike."

I said I wasn't doing anything of the kind, and pointed out to him that his joke, whatever it might be, did not strike me as being particularly jocular from my point of view.

He said, "Doant it, now? Eh, lord, but this beats cockfightin'." Then he went on to say that if I kept round by the four-acre, and crossed the "stooble," and got over the hedge at the end, I'd be "roight agin t' 'ouse." Only I'd best not let my "ant" drop her eye on me, because she was dead against trespassers, and might make it hot for me, even though I was her "nevvy"'—which he with the bread and cheese begged respectfully, "axing your pardon," to doubt.

I was half way over the stubble when a voice called out, "Holloa! you there!"

I looked in the direction the voice came from, and saw sitting on another gate (there seemed a good deal of sitting on gates going on down in those parts) a little girl about ten years old, in a large bonnet, with stout lace-up boots and black stockinged legs, shapely withal, one of which dangled and swung, whilst the other was locked in between the bars to steady its owner. "Halloa?" went on she; "what are you doing on my field?"

"Halloa!" I said; "who are you?"

"I'm Miss Ann Man," she answered; "who are you?"

"I'm your nevvy!"

I cannot deny that I was rather annoyed with my Aunt Ann for being so unnaturally young, but perhaps it was not altogether her fault. I rather attribute the blame to my plaguey tough maternal grandfather, who went marrying again, and got to be a parent at the age of seventy odd. No, I didn't exactly owe Aunty Ann any grudge on her own account, but it was, you must allow, just a little bit annoying when I came to reflect how I had signed the letter I wrote to her announcing my arrival—"your obedient and dutiful nephew."

"There, now," said she, "I told Dorothy you couldn't be a little boy, or you wouldn't have wrote so well; and I wish you'd been a little boy, because we'd have played games, and rode on my pony, and gone a-bird-nesting. You can't play games, can you? Can you climb trees? Your legs are too long for the pony; you'd have to walk both sides of him."

The Dorothea Aunt Ann spoke of was an elderly female who had been my maternal grandmother's aunt, and the name of the party who had choked himself on the gate was Job. Presently an Isaac and a Robin came to light, and this made up aunty's household, over which aunty ruled supreme. Her "nevvy" by the name of Oldboy (I was called "old boy" right off, and kept to it, as the most appropriate appellation obtainable) took up his residence with her also, and occupied a room in a gable end which had not three square yards of it anywhere that was not on the slope, or the slant, or the "skewwhiffle," and here, like some elderly jackdaw that had travelled and seen many things the knowledge of which was of no particular service to him afterwards, I sat and wagged my head, and watched wistfully the old things being done that I had well nigh forgotten, picking up my lost knowledge of them as best I could. Among other things, I picked up marbles and rounders. I never got quite up to "nesties," the climbing being a little beyond me, but I picked up cribbage more easily, being next best at it after Dorothea, and much stronger than Job.

"Oh, you are a butter-fingers!" said Aunty Ann one day. I never could catch a ball in my life, and it was in reference to this failing on my part. "I say, Oldboy, continued she, "I wonder how ever you made all your money out at Barbadoes?"

There is no disputing the fact: my Aunt Ann had formed but a mean estimate of my abilities in sports and pastimes. She herself, when she pegged, scarcely played cribbage as fairly as she might have done—I will always maintain that; and her bowling was of an uncertain and vexatious nature a short-sighted person stood little chance against. That pony that has been alluded to, little as it was, managed to throw me. I gave Job, who looked on and choked himself, a shilling not to tell aunty about it, but the fellow blabbed, I believe, because Aunt Ann said later in the evening, apropos of nothing, "They've no horses out at Barbadoes, I suppose?"

One day she asked me, suddenly, "Can you sing?" and I saying "A little, she ordered me to sing at once; and, when I had got through half a verse, to stop again, without assigning any reason. Another day she said, "Nevvy, do you ever say your prayers?"

I answered with some confusion, "Of course I do."

"Let's hear you say them," she said; and I think that moment was as awkward a one as I ever had in my life.

After looking at me a long while, on one occasion, she asked suddenly, "What do you mean to do with all your money, Oldboy?"

"I shall leave it to you in my will."

"Where will you go then, when you've left it? To Barbadoes?"

"Not to such a hot place as Barbadoes, I hope," said I; "I shall die, you know."

"People before they die ought to make their wills, oughtn't they? And I've money of my own, too. I ought to make *my* will, oughtn't I, Oldboy? Will you show me how?"

When I that day showed Aunt Ann how, and, together, we drew up a formal document, bestowing the farm and the fields and the pony and a tame rabbit in a perfectly equitable manner among Dorothea. Job, Isaac, and Robin, and had after that made sundry and divers alterations and amendments, in the shape of codicils, Aunt Ann stopped suddenly and exclaimed, "Supposing, Oldboy, I die first. What's to become of you when you've left me all your money, and I've given everything away?"

This problem puzzled her not a little, and I found it difficult to explain. So Aunt Ann signed and sealed her instrument with a doubtful shake of the head; and I, like a big fool as I was, duly witnessed it, thinking it (bigger fool still) a great joke. Joke, indeed! Heaven save the mark, that was the grimmest joke I ever played a part in.

"Old men's children seldom make old bones," Dorothea has many times said—as if she knew, though! It's as foolish as many another old woman's saying old women gabble over again and again.

Merely a child's ailment, the doctor tells us, but adds it is often enough fatal in its effects. Will it be so this time? There is scarcely a sound in the half-deserted room where Dorothea and I sit silent watching; and our little darling lies silent too with closed eyes. Scarcely a sound in the house, except the ticking of the clock upon the stairs, and the striking of the hours when one by one they reach an end. Scarcely a sound in the country without, on which just now the sun has slowly set, and the twilight is creeping stealthily, presently to close in on us and thicken into blackest night.

Upon a peg behind the door the big bonnet hangs, and it is so long since she had it on. The little leather hob-nailed boots were polished well nigh three months ago, and set out ready for her, but she has not worn them yet. The little wasted hand that used to bowl me out so royally and cheat me so deftly at cribbage lies here listless in mine. It moves now ever so feebly. The doctor said at this hour we might expect a change for the better or worse, he could not say.

Another flutter of the slender fingers, and then they tighten over mine, and her eyes open wide.

"Which is it, Dorothea? Quick, quick."

Oh, kind heaven, spare her. She is so young—so pretty—so good, and I am so lonely. This is all I have left to love. Do not take it from me.

What were the prayers I used to say when I said prayers? Would it be of any use to say them now? Hush—hush! *she* is saying her prayers.

"Pray God makes me well and strong again once more."

And God hears her.

The danger is all past and gone now, and Aunty sits upon my knee, and looks up into my withered face smiling:

"Don't cry, Oldboy!"

